# Novel approaches targeting ferroptosis in treatment of glioma

**DOI:** 10.3389/fneur.2023.1292160

**Published:** 2023-11-06

**Authors:** Jing Zhao, Fengling Zang, Xiaoya Huo, Shengzhe Zheng

**Affiliations:** Department of Neurology, Affiliated Hospital of Yanbian University, Yanbian Korean Autonomous Prefecture, Jilin, China

**Keywords:** glutathione peroxidase 4, natural compounds, cancer, reactive oxygen species, erastin

## Abstract

Glioma is a malignant brain tumor with a high mortality rate; hence novel treatment approaches are being explored to improve patient outcomes. Ferroptosis, a newly described form of regulated cell death, is emerging as a potential therapeutic target in glioma. Ferroptosis is characterized by the accumulation of lipid peroxides due to a loss of intracellular antioxidant systems represented by the depletion of glutathione and decreased activity of glutathione peroxidase 4 (GPX4). Since glioma cells have a high demand for iron and lipid metabolism, modulation of ferroptosis may represent a promising therapeutic approach for this malignancy. Recent studies indicate that ferroptosis inducers like erastin and RSL3 display potent anticancer activity in a glioma model. In addition, therapeutic strategies, including GPX4 targeting, lipid metabolism modulation, inhibition of amino acid transporters, and ferroptosis targeting natural compounds, have shown positive results in preclinical studies. This review will provide an overview of the functions of ferroptosis in glioma and its potential as a suitable target for glioma therapy.

## Introduction

Glioma is a highly malignant and aggressive brain tumor that originates from glial cells, which are the support cells of the brain (1). Historically, glioma has been classified according to the cell type, grade, and location within the brain. However, the recent advances in molecular profiling have revealed that gliomas are highly heterogeneous tumors with distinct molecular subtypes, which have different clinical behaviors and treatment responses (2). The pathogenesis of glioma is complex and multifactorial, involving genetic and environmental factors, as well as epigenetic and microenvironmental influences (3). The most common genetic alterations observed in glioma are mutations in isocitrate dehydrogenase (IDH1/2) (4), TP53 (5), and ATRX (6), as well as amplification of EGFR (7) and loss of PTEN (8). These mutations affect various cellular pathways, such as cell cycle regulation, DNA repair, and signaling, and contribute to the development and progression of glioma. In addition, epigenetic modifications, such as DNA methylation and histone alterations, as well as microenvironmental factors, such as immune cells, blood vessels, and extracellular matrix, play important roles in glioma pathogenesis (3, 9).

The current standard of care for glioma includes surgical resection, radiation therapy, and chemotherapy with temozolomide (TMZ) (10, 11). However, despite aggressive multimodal therapy, glioma remains a highly lethal disease with a median survival of less than two years for glioblastoma (12), the most common and aggressive subtype of glioma. The dilemma of glioma therapy lies in the limited efficacy of current treatments, as well as the potential toxicity and side effects associated with these treatments. Overall, glioma is a complex and challenging disease that requires a better understanding of its molecular pathogenesis and the development of novel therapeutic strategies.

### Ferroptosis in glioma

Ferroptosis is a type of programmed cell death that is distinct from apoptosis, necrosis, and autophagy (13). It is initiated by the accumulation of lipid hydroperoxides, which results from uncontrolled lipid peroxidation in the plasma membrane (13, 14). Ferroptosis is dependent on iron and is regulated by various molecular mechanisms, including glutathione peroxidase 4 (GPX4), lipid metabolism, and the cystine/glutamate antiporter system (XC-) (14). GPX4 is a critical regulator of ferroptosis, as it catalyzes the reduction of lipid hydroperoxides and prevents the buildup of reactive oxygen species (ROS) (14). Dysregulation of GPX4 has been implicated in several types of cancer, including glioma (15).

Glioma cells have been shown to undergo ferroptosis in response to various stimuli, including chemotherapeutic agents, radiation, and nutrient deprivation (16–18). However, glioma cells are also capable of developing resistance to ferroptosis through various mechanisms, including the upregulation of antioxidant systems, altered iron metabolism, and alterations in the XC- system (19). Glioma cells are known to have high levels of antioxidants, such as glutathione and NADPH (20), which can counteract the increase in ROS levels that initiate ferroptosis. Additionally, glioma cells can upregulate the expression of transferrin receptor 1 (TfR1) and ferritin (21, 22), which are involved in iron uptake and storage, respectively. Increased expression of the XC- system is another mechanism by which glioma cells can evade ferroptosis (23, 24). The XC- system is responsible for the uptake of cystine, which is converted to cysteine and used in the production of glutathione (25). The dysregulation of the XC- system has been linked to resistance to ferroptosis in glioma cells.

The dysregulation of ferroptosis in glioma is a critical factor in the development and progression of this aggressive cancer. While the mechanisms underlying this dysregulation remain complex and multifaceted, the potential role of ferroptosis as a therapeutic target for glioma is a promising area of research. Further studies are needed to elucidate the molecular mechanisms that underlie the dysregulation of ferroptosis and to develop novel drugs that target this process in glioma.

### Recent advances in novel approaches targeting ferroptosis in glioma

Several studies have suggested that targeting the key components of ferroptotic pathway may be a potential therapeutic strategy for the treatment of glioma (19). For example, the inhibition of GPX4 has been shown to induce ferroptosis in glioma cells and enhance the cytotoxicity of chemotherapeutic agents. Similarly, targeting the XC- system has also been proposed as a potential therapeutic strategy for the treatment of glioma, as it could enhance the sensitivity of glioma cells to ferroptosis.

#### GPX4 targeting

GPX4 is a key enzyme in the reduction of lipid peroxides, and its inactivation is a crucial step in the induction of ferroptosis. Numerous small molecule inhibitors have been developed and investigated for their ability to inhibit GPX4 and induce ferroptosis in various cancer types, including glioma (26). One of the most studied GPX4 inhibitors is RSL3, which selectively binds to and inactivates GPX4, leading to an accumulation of lipid peroxides and subsequent ferroptotic cell death (27). Preclinical studies have shown that treatment with RSL3 results in a significant reduction in glioma cell viability and tumor growth inhibition (27). Another GPX4 inhibitor, ML162, effectively binds to GPX4, inhibiting its enzymatic activity and causing ferroptotic cell death (28). However, there are no evidence have shown that ML162 sensitizes glioma cells to ferroptosis. FIN56 is another novel inhibitor of GPX4 that promotes GPX4 degradation and induces tumor ferroptosis. In multiple tumors including glioma, FIN56 has been shown to significantly decrease GPX4 expression, increase intracellular peroxide levels, induce cell ferroptosis, and effectively inhibit tumor cell proliferation (29–32). In addition, FIN56 can also activate squalene synthase to promote coenzyme Q10 depletion independent of GPX4 degradation (33). Coenzyme Q10 is an essential cofactor in the mitochondrial respiratory chain that protects cells from oxidative stress damage (34). Depletion of coenzyme Q10 by FIN56 potentially disrupts mitochondrial function and causes mitochondrial iron overload, leading to ferroptosis induction (35).

#### Lipid metabolism modulation

Alterations in lipid metabolism can influence the susceptibility of glioma cells to ferroptosis. Certain metabolic enzymes involved in lipid biosynthesis and metabolism, such as acyl-CoA synthetase long-chain family member 4 (ACSL4) and stearoyl-CoA desaturase 1 (SCD1), have been identified as potential targets (36). ACSL4 plays a crucial role in the synthesis of polyunsaturated fatty acids (PUFAs), which are precursors of lipid peroxidation (37). Inhibition of ACSL4 decreases the incorporation of PUFAs into membrane lipids, leading to reduced lipid peroxidation and ferroptosis resistance in glioma cells (38). Similarly, SCD1 inhibition impairs the synthesis of monounsaturated fatty acids (MUFAs), promoting temozolomide (TMZ) sensitivity in glioma cells (39).

#### Iron nanoparticles

Recent studies have shown that Iron nanoparticles offer a promising means to induce ferroptosis selectively in glioma cells due to their unique physicochemical properties and ability to modulate iron metabolism. The combination of iron nanoparticles with other therapeutic agents, such as small interfering RNA (siRNA) and cisplatin, has demonstrated enhanced efficacy in selectively inducing ferroptotic cell death within glioma cells (40, 41). Through the exploitation of iron metabolism, this approach has the potential to specifically target and eradicate malignant glioma cells while sparing normal cells, thereby reducing systemic toxicity often associated with traditional therapies. The multifaceted nature of this novel treatment approach extends beyond therapeutic delivery and ferroptosis induction. Iron nanoparticles offer additional benefits, such as their capacity to serve as contrast agents for magnetic resonance imaging (MRI) (42). This dual functionality allows for both diagnosis and treatment monitoring, providing a comprehensive framework for personalized glioma management.

#### Inhibition of amino acid transporters

Amino acid transporters, such as system x_c^– and solute carrier (SLC) 7A11, are essential for the uptake of cystine and other amino acids required for the synthesis of glutathione and other antioxidants. Recent studies have shown that inhibition of amino acid transporters, such as system x_c^– and SLC7A11, can induce ferroptosis in glioma cells and suppress tumor growth in glioma models (43).

#### Natural chemicals targeting ferroptosis

The investigation of natural compounds for their potential in targeting ferroptosis in the treatment of glioma is an intriguing and promising area of research. With the rising interest in alternative and complementary approaches to conventional cancer therapies, exploring the therapeutic potential of natural compounds is crucial. Natural compounds such as curcumin, fucoxanthin, terpinen-4-ol, Boric acid and dihydroartemisinin, have been found to possess remarkable anti-cancer properties, including the ability to induce ferroptosis (44–48). These compounds target ferroptosis through various mechanisms, including iron metabolism, glutathione peroxidase 4, and the cystine/glutamate antiporter system. By targeting the intricate mechanisms involved in ferroptosis, natural compounds offer a potential avenue for developing novel and less toxic glioma treatment options.

In addition to their ability to trigger ferroptosis, natural compounds often possess multiple other beneficial properties, such as anti-inflammatory and anti-proliferative effects (49). This could offer additional advantages in the treatment of glioma. One of the advantages of natural compounds is their relatively low toxicity compared to traditional chemotherapeutic agents, making them potentially suitable for combination therapy or as adjuvants alongside conventional treatments. Furthermore, the abundance and accessibility of these compounds can make them more cost-effective and widely available for patients who may benefit from them.

Overall, the exploration of natural compounds targeting ferroptosis in glioma treatment represents an exciting frontier in cancer research. Harnessing the power of nature’s resources has the potential to revolutionize treatment options and improve outcomes for patients with glioma, but extensive investigation and validation are still needed before these compounds can be incorporated into clinical practice.

## Conclusions and perspectives

In conclusion, ferroptosis has emerged as a new promising therapeutic target for the treatment of glioma. To date, several novel approaches targeting ferroptosis have been identified, including natural compounds and small-molecule inhibitors of specific pathways involved in ferroptosis regulation. *In vitro* and *in vivo* experiments have shown these approaches to have significant anti-tumor effects, validating the potential of ferroptosis-based therapy for glioma treatment. However, there are still challenges and limitations that need to be overcome. Firstly, it is important to note that ferroptosis research is still in its early stages, and many of the mechanistic details remain to be fully elucidated. For example, the role of specific proteins such as GPX4 in ferroptosis regulation is still not completely understood. Future studies aimed at decoding ferroptosis regulation will provide valuable insights into the phenomenon, and might identify additional targets for therapeutic intervention. Secondly, glioma is a highly heterogeneous disease, with different subsets of cells often respond differently to treatment. The subset of cells that respond best to ferroptosis-based therapy may be different from those that respond best to traditional therapies. Therefore, a better understanding of glioma heterogeneity will help in designing more effective treatment strategies.

Additionally, the complex interaction between glioma cells and the microenvironment is also a key factor that needs to be taken into account. Glioma cells interact with other cells and signaling molecules in the microenvironment, which can influence their response to ferroptosis-based therapy. Therefore, future research should explore how the microenvironment can be utilized to enhance the effectiveness of ferroptosis-based therapy. Toxicity also remains a key consideration for any potential therapy. It would be helpful to explore the toxicological profile of ferroptosis inducers in greater detail, including their toxic effects on normal cells, as well as any potential cumulative effects after long-term treatment. Lastly, ferroptosis-based therapy may also need to be combined with other modalities of treatment to optimize its effectiveness. This could include combining ferroptosis-based therapy with radiation therapy or chemotherapy, or utilizing it in combination with immune checkpoint inhibitors or other immunotherapeutic approaches. A more comprehensive exploration of ferroptosis-based therapy’s limitations and challenges would assist in overcoming these obstacles and bring us closer to developing new treatment options for this difficult-to-treat disease.

## Author contributions

JZ: Writing – original draft. FZ: Writing – original draft. XH: Writing – original draft. SZ: Writing – original draft.
